# DPA: Deep Parcellation Analysis for Human Trait Prediction

**DOI:** 10.1007/s12021-026-09823-z

**Published:** 2026-11-03

**Authors:** Xiaohe Chen, Zeliang Sun, Chao Huang, Rongjie Liu

**Affiliations:** 1 Department of Statistics, Florida State University, 117 N Woodward Ave, Tallahassee, FL 32304 USA; 2 Department of Epidemiology & Biostatistics, University of Georgia, 101 Buck Rd, Athens, GA 30602 USA; 3 Department of Statistics, University of Georgia, 310 Herty Dr, Athens, GA 30602 USA

**Keywords:** Brain parcellation analyses, Clustering analysis, Fiber autoencoder, Human connectome project, Multiscale feature engineering

## Abstract

Mapping individual differences in white matter organization is a central goal of human brain mapping. Atlas-based structural connectome analyses are widely used but are sensitive to anatomical assumptions and may miss fine-grained connectivity patterns. Data-driven fiber clustering mitigates some limitations but typically relies on streamline endpoints, overlooking rich geometric information along full trajectories. In this paper, we introduce Deep Parcellation Analysis (DPA), a geometry-aware framework for white matter parcellation and trait association. DPA samples multiscale geometric features along entire fiber streamlines and encodes them with a Multiscale Fiber AutoEncoder (MFAE) to produce compact latent representations. These enable clustering that preserves intrinsic fiber geometry independently of anatomical atlases, supporting interpretable mapping of connectivity patterns linked to individual differences. Applied to diffusion magnetic resonance imaging data from the Human Connectome Project Young Adult (HCP-YA) cohort, DPA captures white matter substrates underlying language-related cognitive traits. It achieves comparable or superior predictive performance relative to atlas-based and principal parcellation approaches while revealing biologically plausible fiber pathways. Critically, DPA identifies trait-relevant connections missed by endpoint-based methods, emphasizing the importance of full streamline geometry. Overall, DPA demonstrates that deep, geometry-informed parcellation provides a powerful, interpretable approach for linking white matter organization to behavior, advancing structure–function studies in human brain mapping.

## Introduction

Advances in neuroimaging have enabled the acquisition of high-resolution, whole-brain magnetic resonance imaging (MRI) data from large cohorts, substantially advancing efforts to map the structural organization of the human brain and its relationship to cognition and behavior. A central goal of human brain mapping is to characterize how inter-individual variability in brain structure and connectivity underlies variability in cognitive abilities, behavioral traits, and clinical phenotypes, while maintaining biological interpretability and reproducibility across populations (Bullmore & Sporns, 2009; Fornito et al., 2016; Xu et al., 2025). Large-scale initiatives such as the Human Connectome Project (HCP) have been instrumental in this effort by jointly acquiring multimodal neuroimaging data and rich phenotypic measures, providing a foundational resource for studying structure–behavior relationships at the population level (Van Essen et al., 2013; Glasser et al., 2016).

A common analytical paradigm for linking brain organization to human traits involves two key steps: first, deriving a representation of brain structure or connectivity from neuroimaging data; and second, relating this representation to behavioral or cognitive measures using statistical or machine learning models (Smith et al., 2015; Feng et al., 2018; Wang et al., 2019; Liu et al., 2023). Within this paradigm, brain parcellation plays a critical role by defining the spatial units over which connectivity is summarized and interpreted. Importantly, parcellation choices are not neutral preprocessing steps; rather, they strongly influence the resulting connectomes, statistical sensitivity, and the neurobiological conclusions that can be drawn (Zalesky et al., 2010; Eickhoff et al., 2018).

Atlas-based anatomical parcellation analysis (APA) remains the most widely used strategy for constructing structural connectomes. In APA, the brain is divided into predefined regions of interest (ROIs) based on anatomical atlases, and tractography-derived streamlines are aggregated to estimate inter-regional connectivity (Hagmann et al., 2008; Zhang et al., 2018b). Atlases such as FreeSurferDKT (Klein & Tourville, 2012) offer standardized ROI definitions that facilitate cross-study comparisons. However, accumulating evidence shows that connectivity estimates and downstream structure–trait associations are highly sensitive to the choice of atlas, ROI granularity, and anatomical assumptions, raising concerns about robustness and interpretability (de Reus & Van den Heuvel, 2013; Zalesky et al., 2010; Liu et al., 2023). Moreover, atlas-based ROIs may obscure fine-grained white matter organization and individual variability that are central to understanding brain–behavior relationships.

Motivated by these limitations, data-driven fiber clustering approaches have been proposed to derive white matter parcellations directly from tractography data (Wang et al., 2011; Zhang et al., 2017, 2018a). By grouping streamlines based on similarity rather than predefined anatomical boundaries, these methods better respect the intrinsic geometry of white matter pathways and have demonstrated improved segmentation consistency and test–retest reliability compared to atlas-based parcellations (Zhang et al., 2019). Building on this perspective, Principal Parcellation Analysis (PPA) was introduced as a tractography-based framework that clusters fibers to define data-driven parcellations optimized to explain inter-individual variability and relate structural connectivity to human traits (Liu et al., 2023). PPA represents an important advance toward parcellations that are tailored to trait-relevant white matter organization rather than fixed cortical atlases.

Despite its advantages, PPA relies primarily on streamline endpoint coordinates to perform clustering and construct parcellation representations. While endpoints capture coarse information about cortical terminations, they ignore the rich geometric and topological information encoded along entire fiber trajectories. Prior diffusion MRI studies have shown that properties such as curvature, torsion, spatial extent, and trajectory shape carry meaningful information about white matter development, organization, and inter-individual differences (Garyfallidis et al., 2012; Vázquez et al., 2020; Yeh et al., 2018). Consequently, endpoint-based parcellation may conflate fibers with distinct trajectories but similar cortical endpoints, limiting sensitivity to distributed pathways and integrative circuits that are critical for higher-order cognitive traits.

**Fig. 1:**
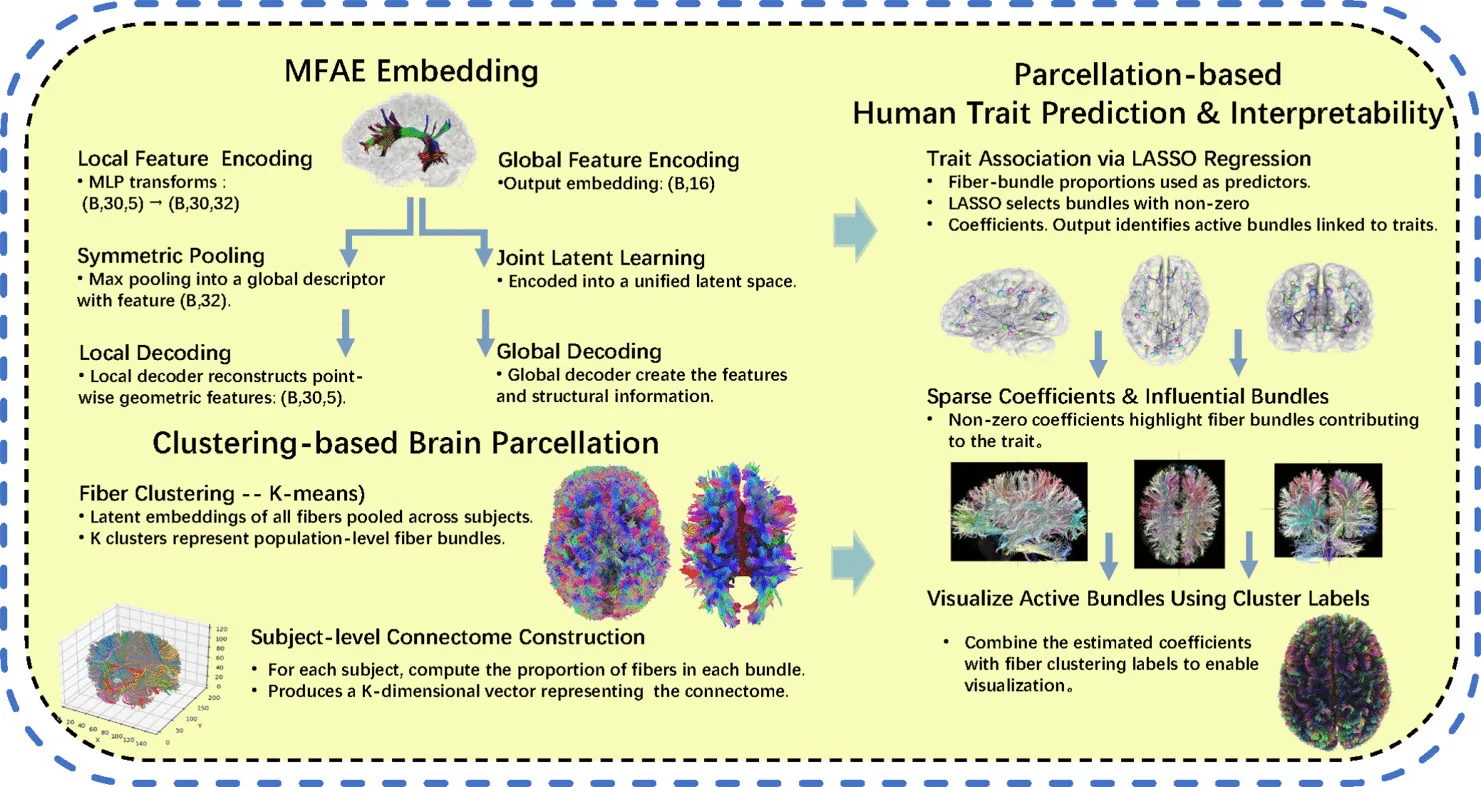
Pipeline of deep parcellation analyses (DPA) for human trait prediction

To address these challenges, in this paper we propose Deep Parcellation Analysis (DPA), a framework that integrates full streamline geometry with deep representation learning to enable biologically informed white matter parcellation and trait association analysis (see Fig. 1). DPA incorporates multiscale geometric features sampled along entire fiber trajectories, capturing both local shape characteristics and global pathway structure. To handle the high dimensionality and nonlinear structure of tractography data, DPA designs a neural network–based autoencoding architecture, Multiscale Fiber AutoEncoder (MFAE), to learn compact latent representations suitable for clustering and downstream analysis (Qi et al., 2017; Han et al., 2019). By this framework, DPA generalizes prior fiber clustering approaches and overcomes key limitations of endpoint-based methods, while remaining fully data-driven and independent of predefined anatomical atlases.

Our work makes several contributions to human brain mapping research. First, our DPA is a novel deep learning–based framework that constructs data-driven white matter parcellations from full streamline geometry, enabling a more faithful representation of white matter organization than atlas-based or endpoint-only approaches. Second, by integrating multiscale geometric information with deep latent representations, our DPA enhances sensitivity to biologically meaningful fiber pathways and supports more interpretable mapping of structure–trait relationships. Third, using diffusion MRI data from the Human Connectome Project Young Adult (HCP-YA) cohort, we demonstrate that DPA reveals distinct fiber connectivity patterns associated with language-related cognitive traits, offering insights into the white matter substrates underlying individual differences. Collectively, these results highlight the potential of deep, geometry-aware parcellation strategies to advance the interpretability and robustness of connectome-based human brain mapping.

The remainder of the paper is organized as follows. Section Materials describes the brain imaging and human trait data used in this study. Section Methods presents the proposed DPA framework. In Section Results, we compare DPA with its ablated variants, PPA, and other state-of-the-art atlas-based parcellation methods in terms of predictive performance, visualization, and interpretability. Finally, Section Discussion & Conclusion provides a discussion and concluding remarks.

## Materials

Data collection and sharing for this project was provided by the MGH-USC Human Connectome Project Young Adult (HCP-YA; Principal Investigators: Bruce Rosen, M.D., Ph.D., Arthur W. Toga, Ph.D., Van J. Weeden, MD) Study. HCP-YA funding was provided by the National Institute of Dental and Craniofacial Research (NIDCR), the National Institute of Mental Health (NIMH), and the National Institute of Neurological Disorders and Stroke (NINDS). HCP data are disseminated by the Laboratory of Neuro Imaging at the University of Southern California.

In this data analysis, we used the same set of 1065 HCP subjects as those in our previous study (Liu et al., 2023), including dMRI data along with human traits, downloaded from the HCP-YA Study. The diffusion MRI data were processed using DSI Studio, a comprehensive tractography platform for diffusion MRI analysis that supports advanced reconstruction and deterministic tracking algorithms (Yeh, 2025). For each subject, diffusion images were reconstructed in their native anatomical space using generalized q-sampling imaging (GQI), a model-free approach that effectively resolves complex fiber orientations and improves tracking accuracy compared to simpler diffusion models (Yeh et al., 2010; Elias et al., 2024; Yeh, 2025). This reconstruction yielded 2-mm isotropic FIB files, which serve as input for deterministic whole-brain fiber tractography implemented in DSI Studio (Yeh et al., 2013a). The tractography procedure produced three-dimensional coordinates of fiber trajectories for all individuals, providing a rich dataset of white-matter pathways for subsequent structural connectivity analyses (Elias et al., 2024). Whole-brain tractography was performed using DSI Studio Hou (Feb 20, 2025) (Yeh, 2025), with 5,000 seeding attempts per subject within an anisotropy-derived brain mask. The step size matched the voxel spacing (2 mm). Angular thresholds were randomly selected between $45^{\circ }$ and $90^{\circ }$, and anisotropy stopping thresholds between 0.5 and 0.7 times the subject-specific Otsu threshold. Minimum and maximum streamline length settings were 30 and 200 mm, respectively. No additional anatomical constraints, directional smoothing, or topology-informed pruning were applied. Tractograms were saved in .trk format. For human traits, we focused on two age-adjusted cognitive measures: receptive vocabulary (PicVocab) and oral reading ability (ReadEng). Brief descriptions of these traits are provided below:

- *PicVocab*. Derived from a picture vocabulary test in which participants hear an audio recording of a word and select, from four photographic images displayed on a computer screen, the image that best matches the word’s meaning.
- *ReadEng*. Obtained from an oral reading recognition test that assesses participants’ ability to accurately read and pronounce letters and words.

These measures capture distinct aspects of human cognition and serve as illustrative examples in our analysis. While we focus on two task-based scores here, the proposed methodology is broadly applicable to other task-based measures or, more generally, to any quantitative traits reflecting human cognitive or behavioral characteristics.

## Methods

Our DPA pipeline consists of three main stages: (1) multiscale fiber feature engineering, (2) clustering-based brain parcellation, and (3) parcellation-based human trait prediction. An overview of the pipeline is shown in Fig. 1.

Suppose fiber trajectories are extracted from *N* mutually uncorrelated subjects. The *j*-th fiber streamline of the *i*-th subject consists of $M_{ij}$ spatial points *(i = 1, … , N)*. Because $M_{ij}$ varies across streamlines, we resample all fibers to a common resolution. Specifically, each streamline is represented by the coordinates of *M* points equally spaced along its arc length. In practice, we set *M = 30* for all streamlines, yielding a uniform representation across subjects and fibers.

**Fig. 2:**
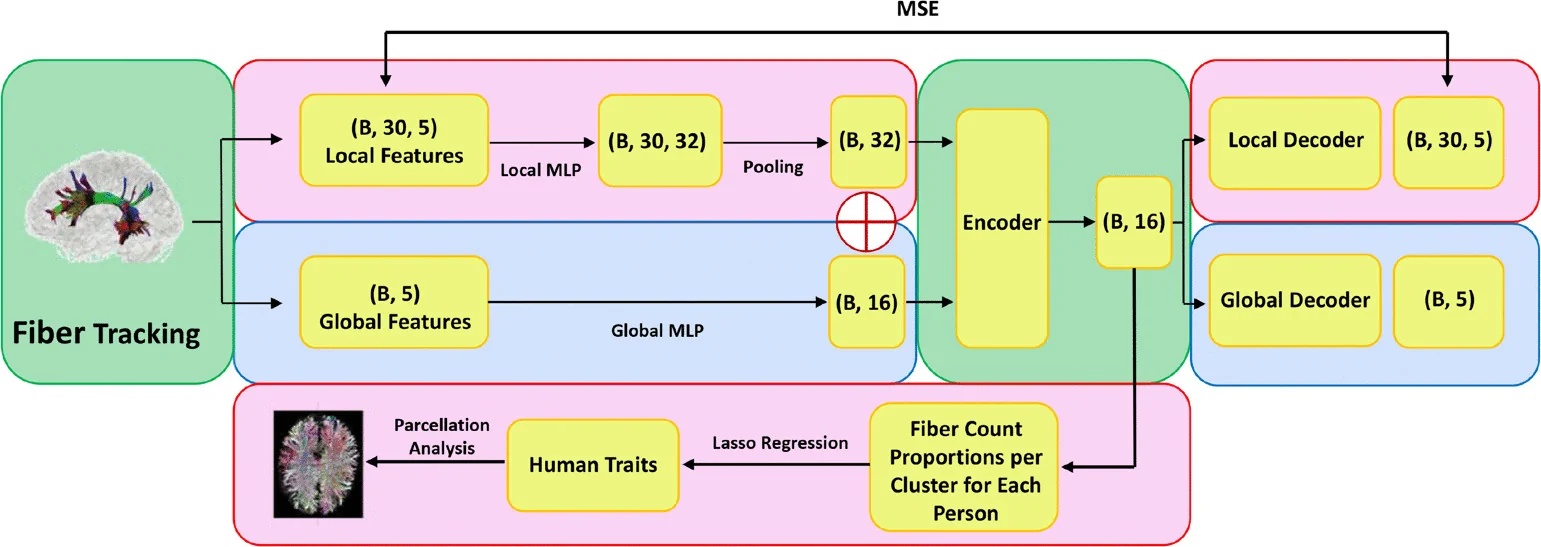
Illustration of multiscale fiber autoencoder (MFAE) architecture

In Step 1, we extract multiscale features from fiber trajectories at both local and global levels. Locally, we compute geometric features at each point, including the 3D coordinates $\textbf{r}_k \in \mathbb {R}^3$, curvature $\kappa _k$, and torsion $\tau _k$, summarized as $\textbf{u}_k = (\textbf{r}_k^\top , \kappa _k, \tau _k)^\top$, *k = 1, … , M*. Globally, we extract fiber-level descriptors, including the fiber center $\textbf{c} \in \mathbb {R}^3$, Frobenius norm *F*, and arc length *L*, collected as $\textbf{v} = (\textbf{c}^\top , F, L)^\top$. To learn informative representations from multiscale fiber features, we propose a *multiscale fiber autoencoder* (MFAE), consisting of an encoder and a decoder (Fig. 2). The encoder applies a shared local MLP $f_l(\cdot )$ across fiber points to encode local geometric features, aggregates pointwise embeddings via max pooling, and concatenates the result with global features encoded by a global MLP $f_g(\cdot )$; the combined embedding is then mapped to a latent representation $\textbf{z}$ by an encoder network *g(· )*. The decoder comprises a local decoder $h_l(\cdot )$ and a global decoder $h_g(\cdot )$, which reconstruct local and global features from $\textbf{z}_{ij}$, respectively. The MFAE architecture is given by

$$ \text {Encoder: } \textbf{z} = g\!\left( \max _{1 \le k \le M} \{ f_l(\textbf{u}_k) \} \oplus f_g(\textbf{v}) \right) , ~ \text {Decoder: } \widehat{\textbf{u}}_k = h_l(\textbf{z}), \; \widehat{\textbf{v}} = h_g(\textbf{z}), $$

and is trained by minimizing the mean squared error (MSE) between input features and their reconstructions using mini-batches of size *B*.

In Step 2, we apply K-means clustering to group fiber streamlines into *K* fiber bundles for brain parcellation Prediction performance was compared across candidate values of *K*, with each value evaluated separately in the HCP analysis. Clustering is performed on pooled fibers from all subjects. For a given *K*, an individual’s connectome is represented by the proportions of their fibers assigned to each population-level bundle. Specifically, the *i*-th individual is characterized by the *K*-dimensional compositional vector $\textbf{w}_i = (w_{i1}, w_{i2}, \ldots , w_{iK}),$ where $w_{ik}$ is the proportion of fibers in the *k*-th bundle (*k = 1, … , K*). This representation can be used for downstream analyses such as trait regression. Importantly, the framework does not require every cluster to be present in each individual, as some individuals may have zero fibers in certain bundles (Liu et al., 2023).

In Step 3, we use LASSO regression, a high-dimensional supervised learning method, to model the association between a human trait $y_i$ and the fiber bundle representation $\textbf{w}_i$. To address the collinearity inherent in compositional data ($\sum _{k=1}^{K} w_{ik} = 1$), we select the bundle with the smallest variance as the reference (here, the *K*-th bundle without loss of generality). The regression model is

$$ y_i = \beta _0 + \sum _{k=1}^{K-1} w_{ik}\beta _k + \epsilon _i, \qquad \widehat{\boldsymbol{\beta }} = \arg \min _{\beta } \left\{ \frac{1}{N} \sum _{i=1}^N \big (y_i - \beta _0 - \sum _{k=1}^{K-1} w_{ik}\beta _k\big )^2 + \lambda \sum _{k=1}^{K-1} |\beta _k| \right\} , $$

where $\boldsymbol{\beta } = (\beta _1, \ldots , \beta _{K-1})^\top$, $\{\epsilon _i\}_{i=1}^N$ are measurement errors, and *λ * is tuned via an inner 5-fold cross-validation within each outer training fold. The $L_1$ penalty encourages sparsity in $\boldsymbol{\beta }$, so many coefficients shrink to zero when *K* is large. Non-zero coefficients highlight fiber bundles contributing to the trait, while zero coefficients indicate bundles with negligible impact. Combining the estimated coefficients with fiber clustering labels enables visualization of the ‘active’ fiber bundles that influence the trait.

In the HCP analysis, MFAE training and clustering were performed using fibers pooled from the full cohort. For each fixed *K*, predictive performance was evaluated using five outer folds. Within each outer training fold, the bundle with the smallest variance across training subjects was selected as the reference, and *λ * was tuned using inner cross validation with five folds. The same reference bundle was used in the corresponding test fold. The models were refitted using the outer training sets, and MSE and $R^2$ were averaged across the outer test folds. Candidate values of *K* were compared using the same outer folds, and the value yielding the best predictive performance was identified from these comparisons. The reported scores therefore assess regression performance under the shared parcellation. Models fitted using the full cohort were used separately for exploratory visualization of fiber bundles.

## Results

### Prediction Performance

To evaluate the predictive performance of our DPA framework, we compared it with several benchmark methods: (i) ablated variants of DPA, including a version based solely on local geometric features (DPA-Local) and a version based solely on global fiber-level descriptors (DPA-Global); (ii) principal parcellation analysis (PPA) (Liu et al., 2023; iii) atlas-based parcellation analysis with Lasso regression (APA-Lasso) (Liu et al., 2023); and (iv) atlas-based symmetric bilinear regression (APA-SBL) (Wang et al., 2019). For the ablated DPA variants, only representations encoded from features at a single scale were used. For the atlas-based approaches, we utilized the FreeSurferDKT cerebral cortex atlas provided by DSI Studio to define regions of interest (ROIs) and compute connectivity matrices. The FreeSurferDKT atlas is manually labeled according to the Desikan–Killiany–Tourville (DKT) protocol (Klein & Tourville, 2012) and comprises 31 cortical regions per hemisphere, yielding 62 cortical regions in total. Based on these connectivity matrices, APA-Lasso and APA-SBL were implemented to predict human traits.

Prediction performance was evaluated using 5-fold cross-validated mean squared error (MSE) and the coefficient of determination ($R^2$) for DPA, PPA, APA-Lasso, and APA-SBL. Lower cross-validated MSE and higher cross-validated $R^2$ indicate that the brain parcellation representations explain a greater proportion of variance in the human traits. In addition, a null model was included as a baseline; this model uses no covariates and predicts traits using only the sample mean.

To assess variability, we conducted an exploratory supplementary comparison of DPA, PPA, and APA-Lasso using 30 repetitions of five-fold cross-validation with different random subject partitions. All three methods used identical outer and inner partitions within each repetition. DPA and PPA parcellations at $K\in \{50,100,150,200,250\}$ were computed from the saved MFAE representation and streamline endpoints, respectively, using identical MiniBatchKMeans settings (n_init=5, max_no_improvement=50, and random_state=0). MFAE was not retrained, and these full-cohort parcellations were held fixed across repetitions. Within each outer training fold, DPA and PPA reference bundles were selected by the smallest variance of raw bundle proportions among training subjects. For all three methods, inner five-fold cross-validation repeated three times selected the preprocessing option (raw or standardized features) and *λ * by minimizing mean inner validation MSE, using 200-point logarithmic paths with $\lambda _{\min }/\lambda _{\max }=10^{-3}$. For DPA and PPA, *K* was jointly selected within the same inner procedure; APA-Lasso used the fixed atlas features. Standardization was fitted within each inner training fold and refitted on the outer training set. Selected regression models were refitted on the outer training sets and evaluated on the corresponding test folds.

For each repetition, MSE and $R^2$ were first averaged across its five outer test folds. We report the mean and sample standard deviation (SD) of these repetition-level scores and their paired differences, defined as $\Delta \textrm{MSE}=\textrm{MSE}_{\textrm{comparison}}-\textrm{MSE}_{\textrm{DPA}}$ and $\Delta R^2=R^2_{\textrm{DPA}}-R^2_{\textrm{comparison}}$. Positive differences favor DPA. The SDs describe variability across random partitions of the same cohort and are not confidence intervals. This assessment is conditional on the shared full-cohort representations and parcellations.

**Fig. 3:**
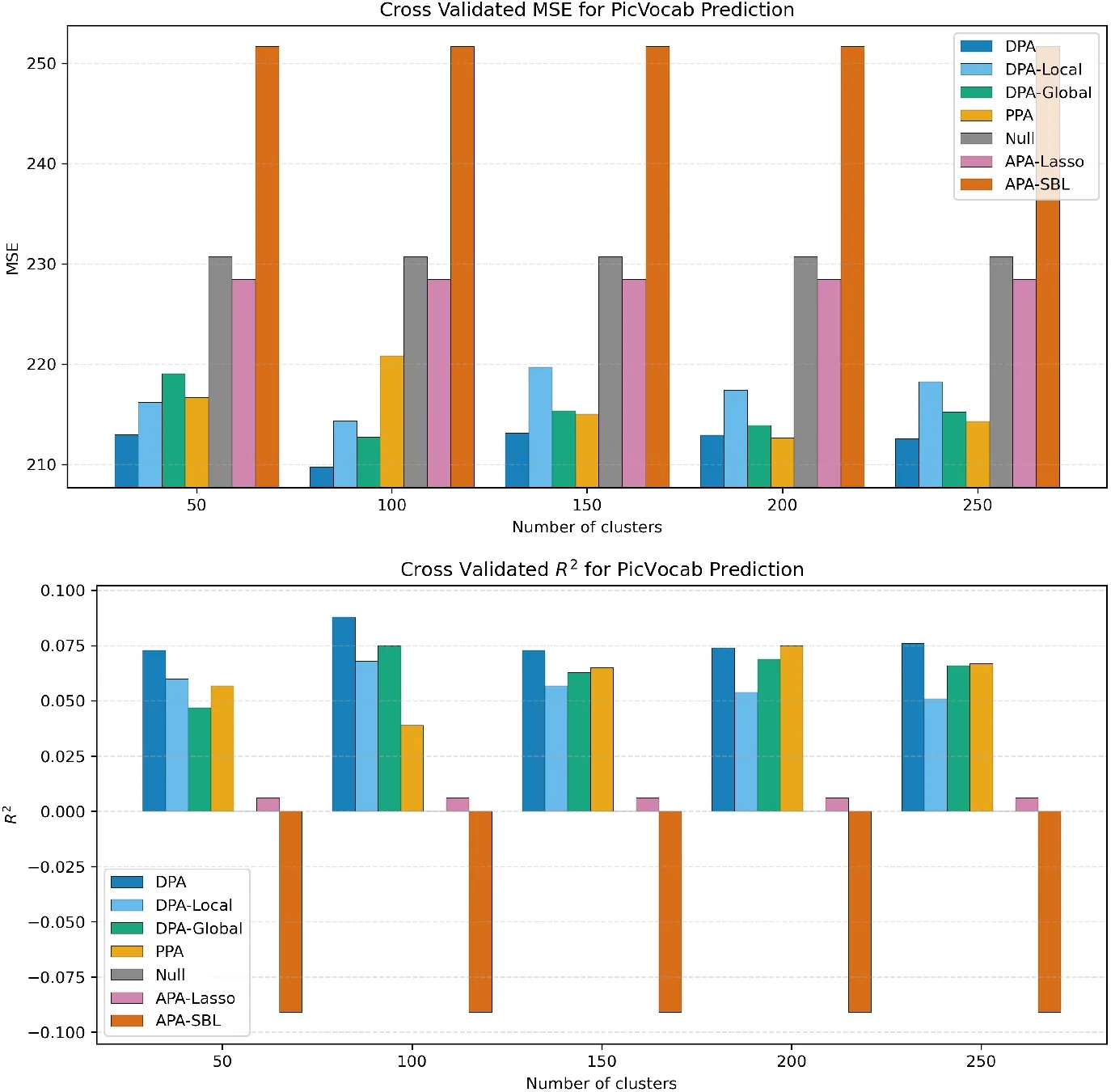
Cross validated MSE (top) and $R^2$ (bottom) for PicVocab Prediction

**Fig. 4:**
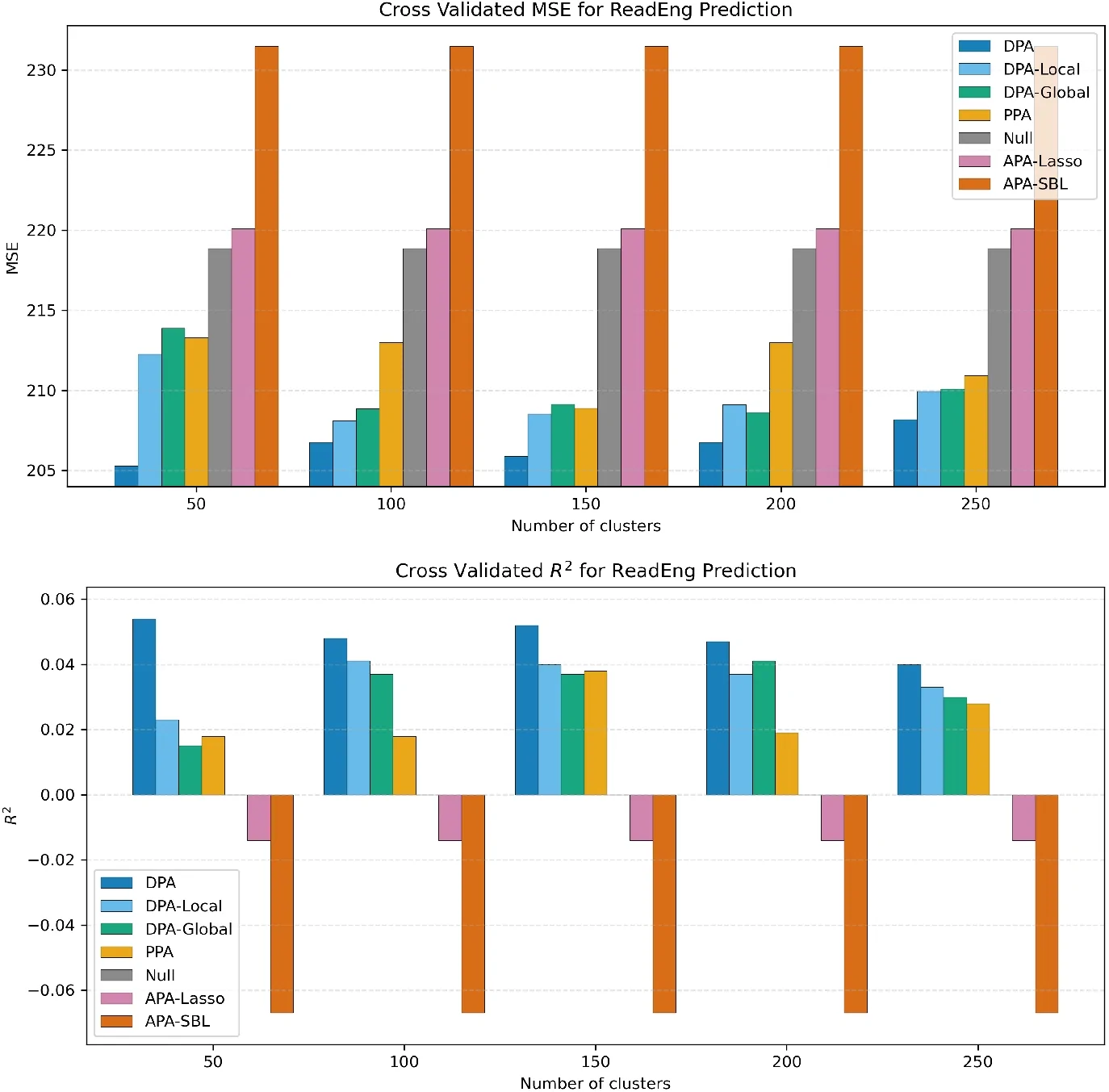
Cross validated MSE (top) and $R^2$ (bottom) for ReadEng prediction

The original prediction comparisons for PicVocab and ReadEng are presented in Figs. 3 and 4, respectively. The APA-based methods use fixed atlas parcellations, and the null baseline uses no parcellation features; their scores therefore do not depend on *K*. In contrast, DPA, DPA-Local, DPA-Global, and PPA depend on the clustering resolution and show changes in predictive performance as *K* varies.

Table 1 summarizes the supplementary repeated evaluation. For PicVocab, DPA had lower average MSE and higher average $R^2$ than PPA, with mean MSEs of 216.54 and 219.48, respectively. The mean paired MSE difference was *2.94± 2.51*, and DPA had lower five-fold mean MSE and higher five-fold mean $R^2$ in 27 of the 30 repetitions. For ReadEng, the corresponding mean MSEs were 209.20 for DPA and 209.85 for PPA, with a mean paired MSE difference of *0.64± 2.30*. DPA had lower five-fold mean MSE and higher five-fold mean $R^2$ than PPA in 17 of the 30 repetitions. Relative to APA-Lasso, DPA had lower five-fold mean MSE and higher five-fold mean $R^2$ in all 30 repetitions for both traits, with mean paired MSE differences of *13.07± 2.12* for PicVocab and *8.25± 2.04* for ReadEng. These descriptive comparisons indicate a more consistent predictive advantage over PPA for PicVocab than for ReadEng under the supplementary evaluation settings.

**Table 1:** Supplementary prediction comparison for 1,065 subjects using 30 repetitions of five-fold cross-validation with identical partitions across methods within each repetition. For DPA and PPA, *K* was selected from * 50, 100, 150, 200, 250* by inner cross-validation; APA-Lasso used a fixed atlas. All three methods used the same inner selection rules for standardization and *λ *. Values are mean ± sample SD of repetition-level five-fold mean scores or their paired differences. Differences are comparison minus DPA for MSE and DPA minus comparison for $R^2$; positive values favor DPA. DPA wins counts repetitions with lower five-fold mean MSE for DPA. The SDs describe partition variability and are not confidence intervals. The full-cohort DPA and PPA parcellations were held fixed across repetitions

Trait	Method	*K*	MSE (mean ± SD)	$R^2$(mean ± SD)	
(a) Prediction performance					
PicVocab	DPA	Inner CV	*216.54± 1.83*	*0.058± 0.008*	
PicVocab	PPA	Inner CV	*219.48± 1.96*	*0.045± 0.009*	
PicVocab	APA-Lasso	Atlas	*229.61± 1.83*	*0.001± 0.009*	
ReadEng	DPA	Inner CV	*209.20± 1.61*	*0.040± 0.008*	
ReadEng	PPA	Inner CV	*209.85± 1.85*	*0.037± 0.009*	
ReadEng	APA-Lasso	Atlas	*217.45± 1.13*	*0.002± 0.006*	
Trait	Comparison	$\Delta \textrm{MSE}$(mean ± SD)	$\Delta R^2$(mean ± SD)	95% CI for$\Delta \textrm{MSE}$	DPA wins (repeats)
(b) Paired differences relative to DPA					
PicVocab	PPA	*2.94± 2.51*	*0.013± 0.011*	*[-3.75, 9.64]*	27/30
PicVocab	APA-Lasso	*13.07± 2.12*	*0.057± 0.009*	*[5.11, 21.03]*	30/30
ReadEng	PPA	*0.64± 2.30*	*0.003± 0.011*	*[-4.80, 6.09]*	17/30
ReadEng	APA-Lasso	*8.25± 2.04*	*0.038± 0.010*	*[1.32, 15.18]*	30/30

Approximate 95% CIs use the Nadeau–Bengio variance correction:$\bar{d} \pm t_{0.975,149} \sqrt{(1/150+1/4)s_{\textrm{fold}}^2}$, where$s_{\textrm{fold}}^2$is the sample variance of the 150 paired fold-level differences. These are pointwise intervals conditional on the shared parcellations and the selected evaluation procedure; they do not adjust for multiple comparisons or procedure selection

Comparing DPA with its ablated variants further highlights the benefit of integrating information across scales. Both DPA-Local and DPA-Global improve upon atlas-based representations and PPA, but neither alone matches the full DPA model. This performance gap suggests that local geometric features and global fiber-level descriptors provide complementary information: local features capture fine-grained microstructural organization, while global descriptors encode long-range connectivity patterns that are critical for human trait prediction. Their joint modeling within the multiscale deep learning framework leads to more informative and expressive brain representations.

In the original comparisons shown in Fig. 4, DPA achieved lower MSE and higher $R^2$ than PPA for ReadEng at all five displayed clustering resolutions (*K=50,100,150,200,250*). The supplementary repeated evaluation with inner selection of *K* also favored DPA on average for ReadEng. These results were obtained under different clustering and parameter-selection settings and are reported separately. The negative average cross-validated $R^2$ values for APA-SBL in the original comparisons indicate limited predictive performance for that method in this setting.

Finally, while both PicVocab and ReadEng benefit from the proposed DPA framework, the absolute $R^2$ values are consistently higher for PicVocab, reflecting potential differences in the strength of structure–function associations across cognitive domains. These results support the predictive value of DPA, with the observed differences between methods varying across traits and evaluation settings.

### Active Fiber Bundle Visualization

**Fig. 5:**
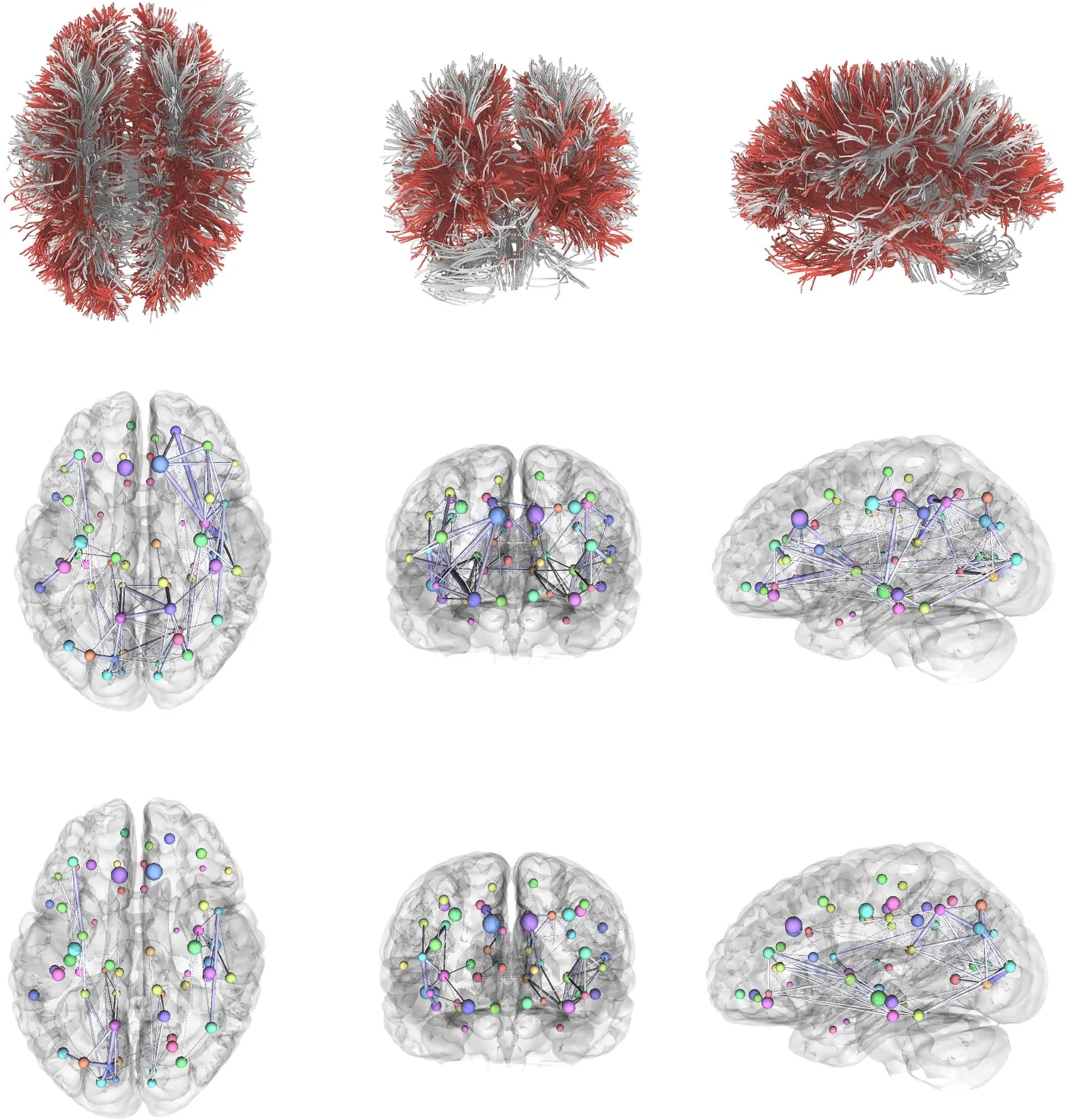
Visualization of active fiber bundles (red) selected for PicVocab given the number of clusters *K=100* (top); the corresponding ROI connectivity graph based on our DPA (middle); and the ROI connectivity graph based on PPA (bottom)

**Fig. 6:**
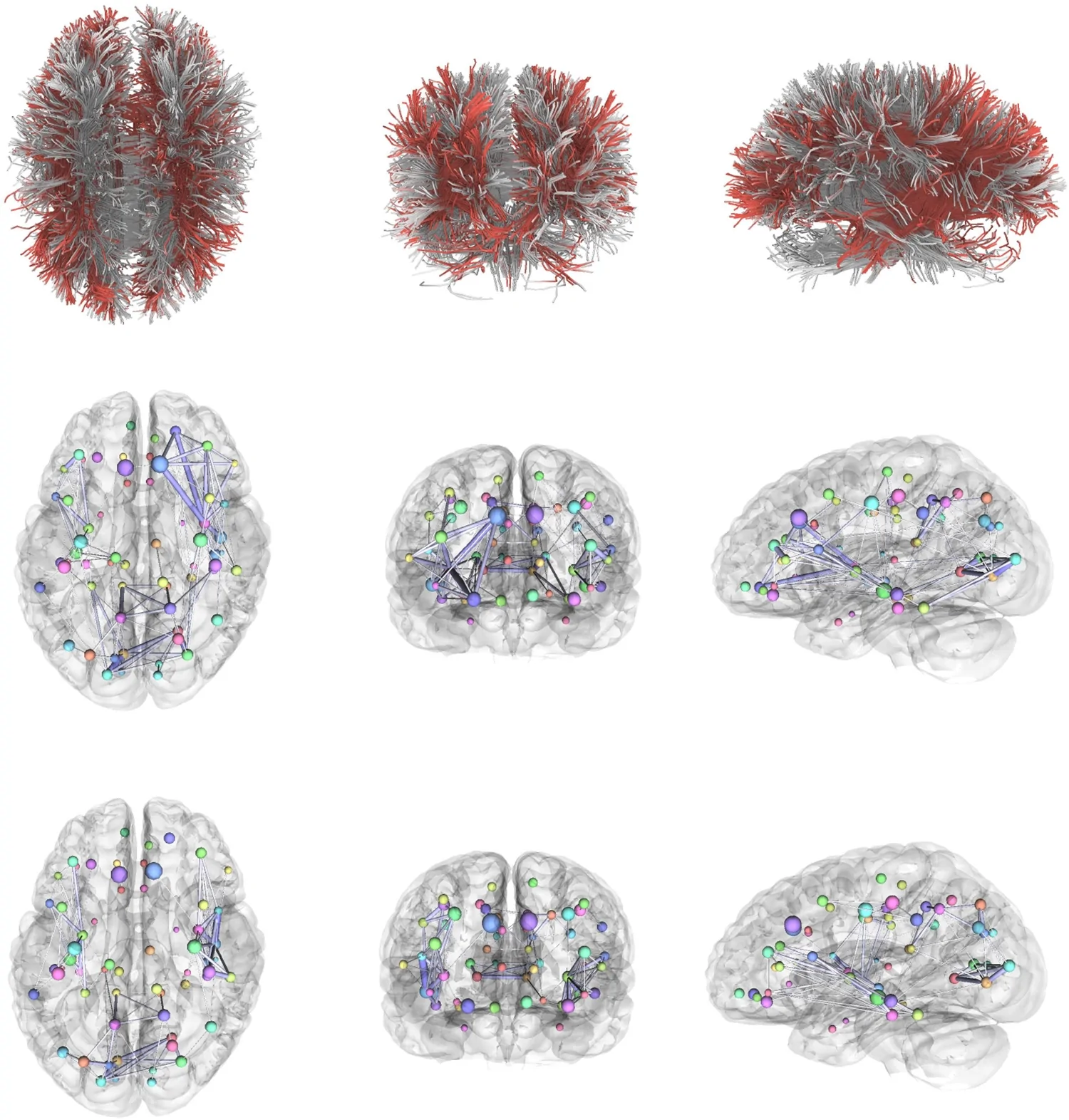
Visualization of active fiber bundles (red) selected for ReadEng given the number of clusters *K=50* (top); the corresponding ROI connectivity graph based on our DPA (middle); and the ROI connectivity graph based on PPA (bottom)

In this section, we present exploratory visualizations of the active fiber bundles at *K=100* for PicVocab and *K=50* for ReadEng, as selected from the original prediction comparisons. The active bundles are shown in the first row of Figs. 5 and 6, respectively. White streamlines denote inactive bundles, whereas colored streamlines denote bundles selected for trait prediction.

To examine the relationship between brain regions and traits, we mapped the identified fiber clusters onto the FreeSurferDKT cortical atlas using DSI Studio. Connectivity graphs were then constructed based on the normalized counts of active fibers connecting each pair of ROIs, as shown in the second row of Figs. 5 and 6. For comparison, the connectivity graphs obtained using PPA with *K = 100* are presented in the bottom row of Figs. 5 and 6.

The active bundle visualizations and atlas mappings involved the bilateral lingual gyri, bilateral lateral occipital cortices, left fusiform gyrus, and left insula. These observations motivate a qualitative discussion of the possible relevance of the selected bundles to the prediction tasks.

One region highlighted in the atlas visualization was the left fusiform gyrus within the ventral occipitotemporal cortex. This region is well known for its role in visual word processing and reading development. Longitudinal evidence indicates that early reading ability predicts subsequent structural changes in the left fusiform gyrus (Beelen et al., 2022), while diffusion MRI studies further demonstrate that the emerging visual word form area exhibits long-range structural connectivity whose microstructural maturation tracks improvements in reading performance during early instruction (Moulton et al., 2019). Functional studies in young children similarly show that print engages a distributed network extending beyond early visual cortex, with individual differences in reading skill reflected in the strength of activation within ventral occipitotemporal systems (Chyl et al., 2023).

The atlas visualization also included connections involving the lingual and lateral occipital cortices. These regions support core visual and visuocognitive processing and are thought to provide essential visual inputs for higher-order language functions. Lesion and neuroimaging studies have linked the lingual gyrus to visual memory and verbal memory performance, underscoring its relevance to language-related tasks that require visual–semantic integration (Kumral et al., 2025). Consistent with this view, the left ventral occipitotemporal cortex has been repeatedly implicated in experience-dependent plasticity for visual word recognition, although its interaction with face-processing systems remains an area of active debate (Rossion & Lochy, 2022).

The atlas mapping also involved the left insula. Previous meta-analytic work has linked anterior insular dysfunction to impairments in speech, cognition, and affective processing (Fathy et al., 2020). These reports provide context for the possible relevance of insular involvement to the prediction tasks.

In the displayed ROI connectivity graphs, DPA and PPA showed differences in the connections associated with the active bundles. In particular, the DPA graphs included right lateral occipital involvement alongside regions such as the lingual gyrus, left fusiform gyrus, and left insula, whereas the PPA graphs mainly highlighted fusiform, inferior temporal, and lingual regions (Liu et al., 2023). These observations describe differences between the bundles selected by the two fitted models; they do not establish that DPA recovered anatomically valid pathways absent from PPA.

The involvement of right lateral occipital regions is qualitatively consistent with previous work on visual and object processing (Rossion et al., 2012; Palejwala et al., 2021). The present biological assessment is based on visualization and comparison with existing literature. It supports qualitative plausibility but does not establish the anatomical accuracy or validity of the resulting bundles. We did not perform independent quantitative anatomical validation or an analysis of trajectories and endpoint distributions to explain the observed differences between DPA and PPA.

### Abaltion Study

First, we examined the sensitivity of DPA to the choice of clustering method by replacing K-means with a Gaussian Mixture Model (GMM) while keeping all other components fixed. As shown in Table 2, the prediction performance obtained using GMM-based clustering is largely comparable to that of the full DPA across different numbers of clusters for both PicVocab and ReadEng. In most settings, the differences in cross-validated MSE and $R^2$ are small, indicating that the performance gains of DPA are not driven by a specific clustering algorithm. This suggests that the learned multiscale fiber representations, rather than the particular choice of K-means, play a dominant role in capturing trait-relevant structural connectivity patterns. In addition, we also assessed the impact of the regression regularization strategy by replacing LASSO with ElasticNet regression (Zou & Hastie, 2005). As reported in Table 2, ElasticNet regression yields performance comparable to LASSO across cluster numbers for both traits, with only minor fluctuations in MSE and $R^2$. These results indicate that DPA‘s predictive performance is robust to the choice of regularization and does not rely critically on sparsity-inducing penalties.

**Table 2:** Ablation study results of DPA for the PicVocab and ReadEng prediction tasks using different clustering and regression strategies

Results for PicVocab			
Number of clusters	DPA Results (MSE, $R^2$)	Ablation Study Results (MSE, $R^2$)	
Clustering Method	Regularization Strategy		
50	212.99 (0.073)	212.52 (0.079)	212.65 (0.075)
100	209.78 (0.088)	210.68 (0.084)	210.23 (0.086)
150	213.14 (0.073)	213.34 (0.072)	213.37 (0.071)
200	212.93 (0.074)	213.74 (0.070)	213.03 (0.072)
250	212.56 (0.076)	212.07 (0.080)	212.60 (0.076)
Results for ReadEng			
Number of clusters	DPA Results (MSE, $R^2$)	Ablation Study Results (MSE, $R^2$)	
Clustering Method	Regularization Strategy		
50	205.30 (0.054)	205.89 (0.050)	206.38 (0.049)
100	206.76 (0.048)	206.62 (0.049)	206.36 (0.050)
150	205.88 (0.052)	205.41 (0.054)	205.96 (0.051)
200	206.76 (0.047)	206.48 (0.048)	206.46 (0.048)
250	208.17 (0.040)	208.78 (0.039)	208.36 (0.040)

## Discussion & Conclusion

We introduced DPA, a framework for human trait prediction using multiscale representations of streamline geometry learned through MFAE. Combining local geometric features with global descriptors enables streamline grouping without predefined cortical ROIs at the clustering stage. This provides a way to explore information along reconstructed trajectories beyond the endpoint representations used by PPA.

The original comparisons suggested a predictive benefit of multiscale geometry, including lower MSE and higher $R^2$ than PPA for ReadEng at the five displayed clustering resolutions. In the supplementary repeated evaluation with inner selection of *K*, DPA had lower average MSE and higher average $R^2$ than both PPA and APA-Lasso for PicVocab and ReadEng. These findings support the predictive value of DPA in the present HCP analysis, particularly for PicVocab, with its relative performance varying across traits and evaluation settings.

The additional value of full streamline geometry may depend on the trait being predicted. PicVocab and ReadEng assess receptive vocabulary and oral reading, respectively, so the structural information useful for prediction may differ. Endpoint locations may already capture some relevant connectivity information, leaving geometric features that are redundant with that representation. Moreover, MFAE is trained to reconstruct streamline features, so its latent representation may retain variation that is weakly related to a particular trait. Noise in estimated fiber orientations can cause tracking errors (Yeh et al., 2013b). Such errors could reduce the predictive usefulness of geometric features. These are possible explanations for differences between traits; the present analyses do not determine which explanation accounts for the observed results.

The findings are also conditional on the tractography pipeline. The GQI reconstruction and deterministic tracking procedure determine the trajectories supplied to MFAE. Choices such as seeding, step size, and angular thresholds may influence tracking performance (Yeh et al., 2013b). In this analysis, the 2-mm reconstruction, 5,000 seeding attempts per subject, and 30–200 mm streamline length limits constrain the geometric detail and trajectories available for parcellation. Changes to these choices may alter bundle proportions and predictive performance. Their influence on the relative performance of DPA and its comparators was not quantified here.

HCP diffusion acquisitions combine high spatial resolution, dense diffusion-direction sampling, and standardized protocols in a predominantly healthy young-adult population (Van Essen et al., 2013). These acquisition conditions may facilitate geometric feature extraction, but the same benefit cannot be assumed for data acquired with lower resolution or different protocols. The restricted age and health profile also limits population generalization, because the distribution of cognitive scores and their associations with brain structure may differ in children, older adults, or clinical populations. No independent cohort or acquisition protocol was evaluated in this study.

The validation design further limits interpretation. MFAE training and clustering used the full cohort before regression cross-validation, so the scores assess regression under a shared parcellation. Performance of the complete pipeline on subjects excluded from representation learning and clustering was not assessed. In the original comparisons, *λ * was tuned within training folds, whereas *K* was compared using the same outer folds and its selection was not nested. Scores at the selected *K* in those comparisons may therefore be optimistic. In the supplementary repeated evaluation, *K*, preprocessing, and *λ * were selected within inner cross-validation. The supplementary SDs describe variability across random partitions of the same cohort. The corrected confidence intervals provide approximate uncertainty estimates conditional on the shared parcellations and selected evaluation procedure, with the intervals for DPA–PPA MSE differences including zero for both traits.

Biological interpretation was limited to qualitative visualization and comparison with prior literature, without independent quantitative validation of bundle anatomy. The selected clusters should therefore be interpreted as features derived from tractography that were associated with trait prediction. Their anatomical validity and the trajectory or endpoint mechanisms underlying differences from PPA remain unverified.

DPA offers a promising approach to exploratory prediction of PicVocab and ReadEng in HCP data. Further validation should prioritize independent cohorts, separation of the complete training pipeline from test subjects, and assessment of sensitivity to tractography choices and parcellation stability before broader conclusions are drawn.

## Data Availability

Data used in the preparation of this article were obtained from the Human Connectome Project (HCP) Young Adult dataset (WU-Minn Consortium; Principal Investigators: David Van Essen and Kamil Ugurbil; 1U54MH091657) funded by the 16 NIH Institutes and Centers that support the NIH Blueprint for Neuroscience Research; and by the McDonnell Center for Systems Neuroscience at Washington University.
